# What can data trusts for health research learn from participatory governance in biobanks?

**DOI:** 10.1136/medethics-2020-107020

**Published:** 2021-03-19

**Authors:** Richard Milne, Annie Sorbie, Mary Dixon-Woods

**Affiliations:** 1 Society and Ethics Research, Wellcome Genome Campus, Cambridge, UK; 2 Department of Public Health and Primary Care, University of Cambridge, Cambridge, UK; 3 Mason Institute for Medicine, Life Sciences and the Law, Edinburgh Law School, University of Edinburgh, Edinburgh, UK; 4 The Healthcare Improvement Studies Institute, University of Cambridge, Cambridge, UK

**Keywords:** informed consent, patient perspective, research ethics, scientific research, general

## Abstract

New models of data governance for health data are a focus of growing interest in an era of challenge to the social licence. In this article, we reflect on what the data trust model, which is founded on principles of participatory governance, can learn from experiences of involving and engagement of members of the public and participants in the governance of large-scale biobanks. We distinguish between upstream and ongoing governance models, showing how they require careful design and operation if they are to deliver on aspirations for deliberation and participation. Drawing on this learning, we identify a set of considerations important to future design for data trusts as they seek to ensure just, proportionate and fair governance. These considerations relate to the timing of involvement of participants, patterns of inclusion and exclusion, and responsiveness to stakeholder involvement and engagement. We emphasise that the evolution of governance models for data should be matched by a commitment to evaluation.

## Introduction

Health research increasingly depends on large-scale data that are either purposefully collected (as in epidemiological studies and infrastructures) or are generated from routine electronic health records.^1^ How to design optimal governance for use of such data resources is a question of increasing relevance in an era of rapid technological innovation and concerns about data privacy, surveillance and public trust.^2 3^ Finding an optimal model of governance for large-scale collections of health data for research purposes is, however, fraught with challenge.

One challenge is that of designing an approach up front that can endure through data collection and analysis over a prolonged period during which the available technologies and prevailing social conditions, law, and norms and expectations may undergo significant change in ways that cannot be anticipated.^4^ A further challenge is that of balancing the potentially divergent goals, interests and rights of the multiple stakeholders, including (but not only) those ‘data donors’ who make personal data about themselves available for research, as well as those who are concerned with the scientific value and outputs of large-scale data endeavours.^5–8^ An often underacknowledged challenge is that of ensuring that governance should be proportionate: it must seek to find ways of recognising and balancing both the risks associated with data use and the counterfactual harms associated with non-use of health data that might otherwise produce collective benefits.^9 10^

A further challenge for governance is its role in securing the trustworthiness of research endeavours, while being transparent about both its operation and limits.^8 11^ In this context, while individual consent remains an important element of governance for health data, it is not, on its own, capable of providing a comprehensive solution to the multiple and potentially competing goals of governance and the other tensions that may arise in relation to data use.^12–14^ Given these complexities, tensions and fragilities, attention has turned to a range of new governance models. A major aim of these models is to secure the broader ‘social licence’ for data use, which experience in the area of *care.data* (an English initiative designed to extract data from primary care medical records for purposes including research), for example, has shown to be precarious and requiring of purposeful design and stewardship.^15^ The concept of a social licence describes how the expectations of society regarding some activities may go *beyond compliance* with the requirements of formal regulation. Failure to fulfil the conditions for the social licence (even if formally compliant) may result in ongoing challenge and contestation.^15^

New governance models for data include, but are not limited to, health information commons, data cooperatives and data trusts.^16–18^ In this article, we propose that much can be learnt for the design and operation of these newer models for governing data from the history of governance in the area of biobanking. Large-scale data resources share many similarities with biobanks^19^: though they both come in many forms, both are premised on the collection of large-scale, persistent data about individuals and their health, and both have sought to innovate with participatory forms of governance. We focus our discussion on the *data trust* model,^20–22^ which has become increasingly prominent in discussion about data governance for research. We begin by briefly outlining the model, highlighting the particular emphasis given to the importance of participation. We then consider how the literature on participatory governance for biobanking might usefully inform debates about data trusts.

## Data trust model

Many new governance models seek to respond to the emerging challenges and vulnerabilities posed by rapid evolution in data technologies that extend (and often reimagine) what can be done with data, the appetite for data from different stakeholders, the twin problems of misuse and underuse of data, and the need to secure trustworthiness. These models may seek closer control over data with the aim of protecting the interests of individuals and groups while also enabling wider availability of data to facilitate the interests of research.^23^ Integral to proposals for such governance models is the introduction of mechanisms whereby individual data donors or groups can exercise the rights granted to them under law to exert control over the sharing and use of data (including data generated through analysis of biological samples) without placing restrictions on data that prevent its use entirely.^18^

In 2017, the UK government report, *Growing the Artificial Intelligence Industry in the UK,* presented the concept of ‘data trusts’ as institutions that might support sharing data in the pursuit of the public interest.^21^ The basic principle underlying the model is that there should be an independent structure for stewardship of data that can enable flexible and inclusive data governance and respect multiple interests.^21 22^ Data trusts are intended to act as independent and sustainable stewards of data focused on sharing data in a fair, safe and equitable way.^21^ They seek to provide inclusive, anticipatory governance of data and make trustworthy decisions about who has access to data, under what conditions, and for whose benefit.^22^ The data trust model can readily be recognised as an example of *participatory governance*, which is characterised by practices of public engagement through deliberative processes.^24^

Several variants of the data trust have now appeared, but they share many common features. Typically, a data trust involves a set of data resources that *a trustee* (more typically, a group of trustees) is required to managed for the benefit of other people (the *beneficiaries*) or for some other purpose than their own.^20 25 26^ Trustees, who may be drawn from a range of backgrounds, take on a stewardship role and are responsible for determining how data should be used.^20 25 27^ Data donors or data subjects—those who put data into a trust and to whom data pertain—may be conceptualised as *settlors*. Settlors may themselves be beneficiaries of the trust,^20^ perhaps along with others (such as those seeking to use the data for research purposes).^25^ A trust *charter* should address its purpose and terms.^22^ This might include matters such as the trust’s guiding ethical principles and the duties of trustees, identification of the beneficiaries and their rights, the rights of settlors, how withdrawal from the trust is managed, and whether trustees can process or share settlors’ data outside the trust.^25^

The data trust model is not one thing, nor is it a single, rigid structure. For instance, in the UK, some envisage data trusts as operating within the legal framework of English trust law,^20^ with data trusts creating a legal relationship that gives rise to particular obligations owed by data trustees to data subjects (as beneficiaries), known as fiduciary duties. Others have explored how different areas of law (eg, contract, commercial or charity law) might facilitate the aims of a data trust model^27^ or tended instead to ‘take inspiration’ from the model of beneficiaries, settlors and trustees (as elaborated previously) in order to formulate a code of governance that may establish a trust’s social licence to operate.^25–27^ Further, a multiplicity of data trusts might exist, allowing data subjects to select the one that most closely aligns with their own goals.^20^ In this article, we do not further consider the contested legal status of data trusts, which is beyond the scope of our discussion.^27^

Whatever form they take, data trusts are intended to have a clear purpose, a legal structure and constitution, trustees and beneficiaries, (some) rights and duties over stewarded data, arrangements for sharing benefits, and sustainable sources of funding and defined decision-making processes.^22^ Delacroix and Lawrence suggest that trusts may further mediate public debate about the relationship between individual benefits and the ‘greater good’.^20^ An especially distinctive characteristic of the data trust model, consistent with the principles of participatory governance, is its emphasis on open and accountable engagement with stakeholders and, in particular, with the people from whom data are collected or to whom it pertains.^20 28 29^ Hence, a UK review of legal and governance considerations related to data trusts highlights the potential for data subjects to be represented as stakeholders in the central governance of a data trust or in an advisory committee.^27^ Exactly what forms engagement, participation and deliberation might take in the context of data trusts, and how to ensure such representation is fair, remains an open question.

It is evident already from early pilot studies and analyses that little about the data trust model is likely to be straightforward.^26 27^ We propose that its development is likely to benefit from learning from other areas that have addressed some of the same questions, including, in particular, earlier attempts to address similar challenges in the area of biobanking.^30^ Biobanking has been similarly characterised by calls to enable people to participate in deliberations on how their contributions can be respected and can contribute to debates about wider societal implications,^5 8 16 31^ but has also experienced challenges in operationalising and achieving these goals.

## Learning from studies of involving and engaging participants and the public in biobanking

In what follows, we provide examples drawn from sources in the scientific literature, study websites and documentation and other published materials about participatory governance for biobanks, primarily focused on the UK and North America. Our aim was not to provide a systematic review of these practices, not least because many are sparsely reported in the scientific and grey literature,^19^ and we did not use formal searches. Instead, we take an *authorial* approach,^32^ drawing broadly on the literature and examples from our own interactions with data governance in the life sciences. We describe initiatives where members of the public and research participants are actively engaged in decision making about the use of data and samples in relatively planned ways, though we recognise that ad hoc consultations may also occur. We examine upstream and ongoing forms of involvement, distinguishing in the latter case between the involvement of individuals or groups, and between community groups and those directly involved in research. We reflect on the implications of each approach for the data trust model, including whether and how they have engaged with tensions around the representation of participants and the wider public.

### Upstream consultations

The defining feature of upstream consultations is that they are conducted when biobank programmes are being planned and established, and are typically motivated by efforts to establish legitimacy and public trust.^31^ Ahead of the establishment of UK Biobank in the mid-2000s, for example, Tutton *et al* 31 argued for the inclusion of representatives of participants in management bodies to foster public trust in the initiative. In practice, upstream engagement in UK Biobank took the form of public consultations on various aspects of the project, including the consent procedure and the ethics and governance framework. These consultations were criticised for failing to open up the core assumptions behind the project or enable members of the public to set the agenda for discussion.^33^

A subsequent wave of biobank engagements have adopted more deliberative approaches, seeking to provide a shared discursive space for public and experts, and have been used widely.^34–37^ Such approaches aim to involve the public in the discussions and resolution of ethical and policy questions, typically by establishing a process and framework within which participants can consider diverse points of view and formulate mutually acceptable solutions or articulate persistent disagreements.^38^ A detailed account of a deliberative approach in practice is provided by the British Columbia Biobank Deliberation (BCBD).^38 39^ The BCBD took place over 2 weekends and involved 25 participants who received information and talks from experts, asked questions and discussed in groups. They were given the task of coming up with design recommendations for a biobank. Facilitators were instructed to tease out disagreements and encouraged participants to elaborate on their views.^38^ The groups reached strong consensus around the value of a biobank and the need for it to have a governing body independent of funders and researchers, although there was more diversity of views on who should sit on this it. This finding is consistent with the other deliberative exercises that have emphasised the need for long-term community oversight to promote trust and to ensure congruence with community values and interests.^36 37 39^

For data trusts, experiences in the field of biobanking suggest that upstream deliberative approaches may be of value in establishing, through stakeholders, the terms and values for a health data trust at the outset. This work may, for example, inform the trust charter, prior to the existence of a defined group of data settlors. The learning from previous work on biobanks, however, also makes it clear that it is important to consider *who* should be included at this stage—particularly in a context where people may have roles as patients, citizens consumers or advocates—and how this may influence discussions and outcomes.^39 40^

### Ongoing involvement

Ongoing involvement may take a number of forms. Here, we consider participant membership of committees and public and participant panels.

#### Participant membership of data access or ethics and governance committees: a shared decision-making approach

An important mode of governance in biobanks (and other large research studies) is that of an ethics and governance or data access committee. Operating at the level of individual projects or across networks of major bioscience initiatives—for example, the National Institutes of Health’s database of Genotypes and Phenotypes and the European Genome-Phenome Archive—access committees aim to ensure fair, efficient, and transparent access to data and samples, while respecting the preferences and values of participants. They may draw on a wide range of expertise and perspectives. For example, the Access Committee of Generation Scotland (GSAC), a large population biobank, includes representation from NHS Research and Development Offices, University Technology Transfer Offices, clinical academics, ethicists and experts in laboratory and data science.^33^ In addition to requests for data access, GSAC discusses issues relating to the welfare of participants, the burden placed on participants by recontact and whether requests for new data may make participants question their health status.

In biobanking, many, if not most, access committees remain expert-led, even where representation from patients and the public is included. A distinctive approach is that of the UK’s Managing Ethico-social, Technical and Administrative issues in Data Access (METADAC) initiative. METADAC is an independent and interdisciplinary panel that makes decisions on data and sample access across eight longitudinal studies.^41 42^ The panel foregrounds the importance of participant-centred decision-making in data access decisions, assessing the extent to which proposed uses of data are commensurate with participants’ expectations and whether the confidence of participants might be put at risk. Although the majority of METADAC members, including the chair, are experts in law, epidemiology, bioethics or sociology, the panel does include representation from people actively involved in studies as participants. At least one of two ‘study-facing members’ must be present for the committee to be quorate. These members bring a participant’s perspective to data access decisions, although they are not participants in the studies for which METADAC provides oversight.

The METADAC approach provides a potentially useful model for data trusts. Its combination of researcher and participant views in decision-making would provide a means of involving the settlors of a data trust in key decision-making processes, an important consideration in maximising the value of data. However, as such efforts expand, clear criteria are needed for the selection of participants and their role in decision-making. The risk otherwise is that settlor involvement in data access committees may become a tokenistic quick fix that neither enables genuine deliberation nor facilitates genuine involvement.

#### Public and participant panels: a consultation and advice approach

Constitution of an independent advisory board within study governance arrangements is an increasingly recommended approach to securing involvement and engagement. One interesting example is that of the Michigan BioTrust, established by the Michigan Department of Community Health (MDCH) to manage a large repository of dried blood spots from neonates held by the state.^43^ While primarily focused on the governance of samples, the MBT is one of few published accounts of the implementation of a charitable trust-based model of biobank governance along the lines proposed by Winickoff and Winickoff.^30^ Discussing UK Biobank, Winickoff and Winickoff argued that a charitable trust would allow the management of the obligations of researchers related to both donated samples and the data derived from them while supporting research in the public interest.^30^ Winickoff subsequently proposed an overall structure for the operation of a biotrust, involving relations between settlors (or donors), beneficiaries and a BioTrust Foundation that would act as the trustee, advised by both an ethics committee and a donor advisory committee.^44^

In the case of the Michigan BioTrust, Chrysler *et al* 43 report that the MDCH was considered to have fiduciary responsibilities to ensure the use of the finite blood spot resource for the benefit of public health, extending to the population from whom blood spots were collected. A management group determines whether any proposed research is a good use of blood samples, guided by a tripartite arrangement of a scientific advisory board, an ethics committee and a community values advisory board (CVAB). This latter represents Michigan citizens and includes representatives from a range of patients’ organisations and civil society groups, as well as genetic counsellors and hospital associations. The purpose of the CVAB, which meets twice a year, is to provide advisory input on the use of samples in research and the governance policies and structure of the BioTrust, and to inform wider community engagement and education strategies. This group and its reports feed into the decision-making process of the MDCH.^45^ However, little detail has been reported on the outcomes or effectiveness of the CVAB involvement.

The governance of the Mayo Clinic biobank similarly includes a citizen‐led Community Advisory Board, constituted of 20 members chosen to reflect the diversity of community interests and backgrounds.^12 37 46^ Its recommendations are not binding, but they are often incorporated into policies and decisions. The board is cochaired by a member of the Mayo Clinic Biomedical Ethics Programme and a community member who is elected by CAB members. Both cochairs are also active voting members of the Biobank’s Biospecimen Trust Oversight Group and Biobank Access Committee. The board has contributed to shaping biobank policy on topics such as incidental research findings and practices for engagement with potential research participants.^12^

Community advisory boards of the type developed at Michigan and Mayo provide a means of incorporating interested public and community groups in the governance of large-scale public bioresources. However, the perspectives they include do not necessarily include those of those who, in a data trust model, might be considered the biobanks’ ‘settlors’—participants themselves. In other studies, ‘participant panels’ have been established to facilitate such direct participant involvement. For example, the governance of the Avon Longitudinal Study of Parents and Children cohort includes an independent ethics and law Committee (ALEC).^47^ This committee comprises clinicians, researchers, people with legal expertise and lay people, including study participants. At least two study participants must be present for the group to be quorate. Further, the decisions of the ALEC are made alongside those of a committee of research participants (and, since 2006, a ‘teenage advisory panel’, established when the child participants were 15 years old). This committee of 30 cohort participants meets every two months to comment on study design, methodology and acceptability for participants.^47^

Various other participant panels or ‘patient forums’ have recently been described.^48 49^ The 100,000 Genomes Project, for example, incorporates a panel of 30 participants meeting three times a year. Its members sit on the data access review committee, the ethics advisory committee and the board of the Genomics England Clinical Interpretation Partnership.^50^ Similarly, the US All of Us Precision Medicine Initiative has made a significant commitment to ‘highly interactive participation’, which aims to transform ‘patients into partners’.^51^ It currently involves a group of 32 participant representatives, 8 of whom sit on the study advisory panel, steering committee and executive committee.^52^

For data trusts, an advice and consultation role for participants through specially constituted groups or panels may be valuable, providing for representation and protection of the interests of people who have a clear stake in decision making about data use. However, it is important that the roles and responsibilities of such groups and panels are clearly articulated, that their impact on decision-making is transparent, and that the time and financial costs associated with involving participant groups in decision making are proportionate with need for input into decision making.

The distinction between participant groups and community groups may be especially important to data trust governance: participants are better able to speak to the risks associated with uses of data provided by them, but also have an interest in the uses of data that may not necessarily align with a ‘public’ interest. Similarly, in the case of a data trust, trustees must manage data in the interests of participants but also consider whether broader engagement may be justified on some issues of societal concern. Balancing these interests will depend to some extent not only on the matter at issue but also on the purpose of the data trust as set out in the trust charter, and the expectations of stakeholders this charter represents—not only of data subjects, but also potentially of both public and private sector groups.

## Discussion

As the capacity to collect data from diverse sources and use them to study and make decisions about health and healthcare grows, it is important that data governance evolves as well. Increasing consensus is emerging that decisions about collecting, sharing and using data should incorporate the perspectives of all stakeholders, including and especially those who provide data.^13^ A system of independent data trusts with differing goals, established according to charters drawn up in consultation with stakeholders to represent their aspirations for data use, may be one architecture for achieving the multiple goals of governance.^20^ In this article, we have shown that learning from experiences in the area of biobanking in the UK and North America offers insights into some of the complexities and challenges in participatory governance for data trusts (box 1). It makes clear that an aspiration to include diverse interests, including those of patients, participants and the public, in governance does not in of itself ensure fair decisions. Data trusts and the principles of participatory governance that inform them are not inherently just^53^: they require careful design and operationalisation.

Box 1 **Recommendations**

- The legitimacy of data trusts relies on open and accountable engagement with stakeholders. These may include both data subjects and the wider public.
- If multiple data trusts exist, the appropriate form of participatory governance will differ, depending on the terms of the trust charter.
- Careful consideration must be given to who is involved, why they are involved, at what stage and for what purpose.
- The data trust model is still evolving and would benefit from rigorous evaluation.

The learning from governance of biobanks highlights several important considerations that are essential to ensure the future development of governance practices for data trusts is fair, effective and proportionate. They relate to the *timing* of involvement of participants, patterns of *inclusion and exclusion*, and *responsiveness* to stakeholder involvement and engagement, and capturing the *impact* of such activities on decisions about data through evaluation. As the plurality of data trusts among which data subjects can choose to settle their data grows—from those that place a heavy emphasis on societal benefits to those that prioritise commercial gains—systematic consideration of these considerations will grow in significance.^20^

With regard to timing, the experiences described previously emphasise that both ‘upstream’ and ‘ongoing’ forms of involvement and engagement have potential value. Prospective deliberation anchors governance in the values and aspirations of the wider population who are expected to support research. As such, it can both contribute to defining what constitutes societal value and establish the trustworthiness of initiatives, providing the basis for a social licence, and will be critical to setting the overall goals, terms and tone of a data trust. Ongoing involvement of participants enables constant reflection and adaptation in response to the experiences of the people most directly affected by research decisions. Data trusts are likely to benefit from both well-designed upstream and ongoing involvement and engagement. Where data trusts either draw on population data or target population-level impacts, but disproportionately rely on data from a smaller group of participants, finding the right balance between upstream and ongoing engagement will be key.

A recognition of the uneven distribution of the benefits and burdens of health research, and the heterogeneity of perspectives, positions and interests that underpin the social licence demands careful design and transparency about who is included in governance and how they come to be included. Biobanking approaches are sometimes criticised for their potential to privilege the perspectives of those involved in research over a civic voice or the rights and interests of minority or excluded groups.^54^ This is particularly challenging for involvement models that focus on individuals or small groups, which may favour those who are well connected, better informed or have performed such roles in the past.^55^ The broader movement to promote patient and public involvement and engagement in health has raised similar concerns.^56^ For example, when critical perspectives have interrogated this movement, they have drawn attention to the need for attention to who is involved and their interests, who gets to speak on behalf of whom, distinctions between patients and the public, and the existence of multiple publics.^56–58^ These are important questions for data trusts. Consistent with principles of fairness and justice, careful and transparent consideration should be given to how decisions about inclusion and representation are reached and with what consequences for those who may be at risk of being underserved, excluded or marginalised; whose voices get heard and with what responses; and whose interests are ultimately served. The role of trustees and the ways in which they make decisions will require careful scrutiny in this regard.

This article has sought to offer learning from the field of biobanking that will be helpful in identifying relevant considerations for participatory governance for data trusts both in the UK and globally. It has some limitations; we did not conduct a systematic search of the literature nor did we engage in formal critical appraisal of individual reports and studies. A striking finding of our work, however, is that the enthusiasm for participatory governance for health research has not been matched by a commitment to rigorous evaluation, meaning that learning from previous efforts (including in the area of biobanking) has not been maximised. What is clear is that relying on participatory governance both as a way of ensuring justice and fairness, and as a way of securing legitimacy or the social licence for data use for research, requires careful design and operationalisation, together with explicit recognition that expectations and aspirations related to data use are diverse and multiple, not a uniform social whole.^59^ Showing how those entrusted with data act in response to the input of stakeholders and in relation to their values is essential to building trust and confidence in data trusts, meaning that evaluation must be centre stage.

## Data Availability

There are no data in this work.
